# Supplementary material for: Structure of a human intramembrane ceramidase explains enzymatic dysfunction found in leukodystrophy
Source: Nat Commun. 2018 Dec 21;9:5437. doi: 10.1038/s41467-018-07864-w (PMC6303388; doi:10.1038/s41467-018-07864-w)
Supplement: Supplementary file 1 — Supplementary Information [file 41467_2018_7864_MOESM1_ESM.pdf]

Supplementary Information

Structure of a human intramembrane ceramidase explains enzymatic dysfunction found in leukodystrophy

Ieva Vasiliauskaité-Brooks[1], Robert D. Healey[1], Pascal Rochaix[1], Julie Saint-Paul[1], Rémy Sounier[1], Claire Grison[1], Thierry Waltrich-Augusto[1], Mathieu Fortier[1], François Hoh[2], Essa M. Saied[3,4], Christoph Arenz[3], Shibom Basu[5], Cédric Leyrat[1]and Sébastien Granier

[1]IGF, University of Montpellier, CNRS, INSERM, Montpellier, France.

[2]CBS, University of Montpellier, CNRS, INSERM, Montpellier, France.

[3]Institute for chemistry, Humboldt-Universität zu Berlin, Brook-Taylor-Str. 2, 12489 Berlin, Germany.

[4]Chemistry Department, Faculty of Science, Suez Canal University, 41522 Ismailia, Egypt

[5]Macromolecular Crystallography, Swiss Light Source, Paul Scherrer Institut, 5232, Villigen PSI, Switzerland

These authors contributed equally: Ieva Vasiliauskaité-Brooks, Robert D. Healey

Correspondence and requests for materials should be addressed to C.L. (email: cedric.leyrat@igf.cnrs.fr) or to S.G. (email: sebastien.granier@igf.cnrs.fr)

Supplementary Table 1. List of primers used for site directed mutagenesis of ACER3-BRIL

Mutant	Forward Primer (5'-3')	Reverse Primer (5'-3')
D19G	CAACGTTGGGTTGGTGTGAAGAGAAC	ATGTGGTAGGTCCCCAATA
E22G	CAACGTTGGATTGGTGTGGAGAGAAC	Same as for D19G mutant
N24G	GTGTGAAGAGGGCTATTCAGTGACTTGG	CAATCCAACGTTGATGTG
E33G	GTATATTGCCGGATTCTGGAATAC	CAAGTCACTGAATAGTTCTC
S99A	GATTTACGCTTGCTGCATCTTCG	ATCGGTAACTCATCCAGTAAC
Y149A	CAAGTCATGGCCGGAATGCTCGTG	GTGGAATATGGGCTCTTTGA
S228A	GACTGGTCTTGGTGCCTATCTCCACA	AGTATGTGCCACCATGCGTG

Supplementary Figure 1.

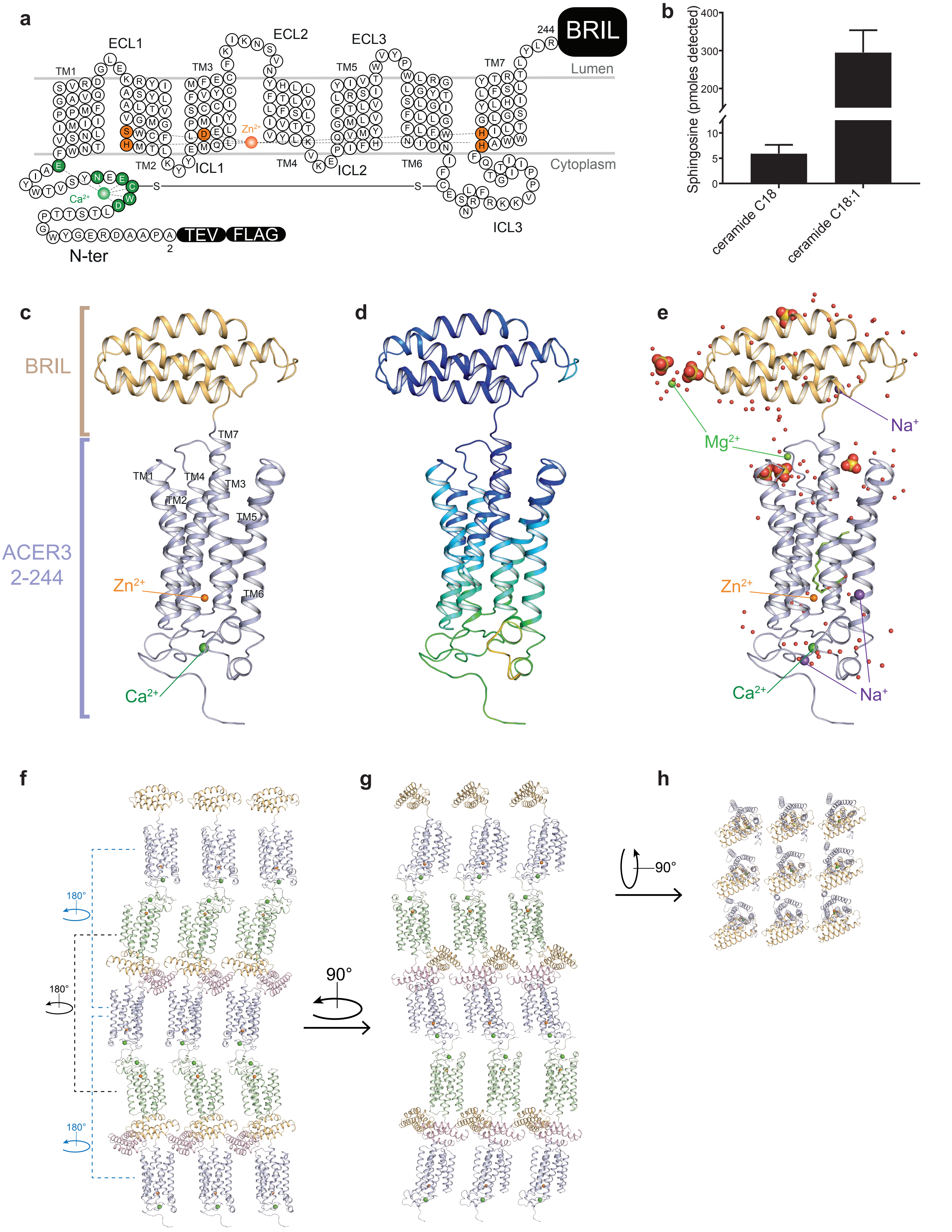

Supplementary Figure 1. ACER3-BRIL crystal structure.

(a) Snake plot presenting the crystallized ACER3-BRIL construct and highlighting the residues coordinating Ca^{2+} and Zn^{2+} (green and orange circles, respectively). The BRIL module fused to the C-teminus as well as N-terminal Flag-tag and TEV cleavage site are shown as black rectangulars. 23 residues at the C-terminal end of ACER3 were truncated to get well diffracting crystals. **(b)** Detected sphingosine in LC-MS/MS analysis revealed that ACER3-BRIL hydrolyzes C18:1 ceramide substrate in a preferred manner over C18:0 ceramide in detergent micelles. Relative sphingosine values are represented as the mean ± SD of three independent measurements performed in six replicates. **(c,e)** ACER3-BRIL crystal structure viewed within the membrane plane with ACER3 and BRIL coloured in light blue and wheat, respectively. **(d)** Cartoon representation of ACER3-BRIL structure coloured according to the crystallographic *B* factors revealing increased flexibility at the N-terminus of ACER3. Regions of lowest and highest B-factor are shown in blue and yellow, respectively. Lattice packing of ACER3-BRIL crystals viewed within the membrane plane **(f, g)** and extracellular side **(h)**. The ACER3-BRIL fusion protein crystallized with an anti-parallel arrangement of the molecules. ACER3 and BRIL are coloured in light blue and wheat or in light green and light purple, respectively.

Supplementary Figure 2.

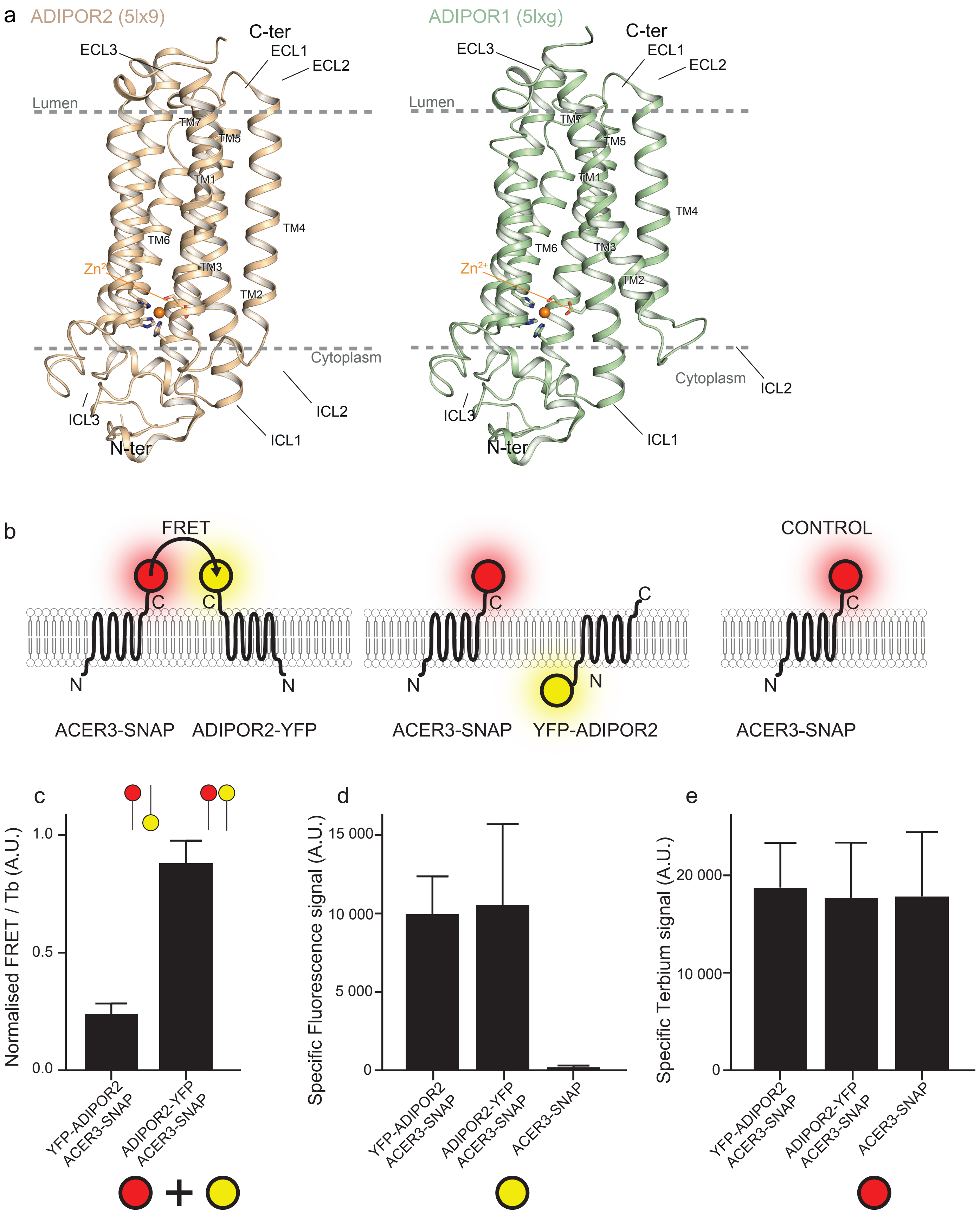

Supplementary Figure 2. Comparisons of the 7TM fold and topology of ACER3 and ADIPORs.

(a) Crystal structures of ADIPOR1 (PDB 5LXG) and ADIPOR2 (PDB 5LX9) shown as light green and dark yellow, respectively, view from the membrane side. This view shows the similar 7TM fold and topology between ACER3 and AdipoRs. **(b)** Schematic representation of the different transfection conditions highlighting the positions of the fluorescent tags used to monitor the topology of ACER3 and ADIPOR2 by Fluorescence Resonance Energy Transfer (FRET) in living cells. **(c)** Graph representing the quantification (as described in the methods section) of the specific FRET normalized by the Terbium signal alone. Signal is higher when ACER 3 and ADIPOR2 have tags in the same orientation. Quantification of specific fluorescence and Terbium signals are represented in **(d)** and **(e)**, respectively, and do not show changes between conditions. The results shown are the mean ± sem of three independent experiments performed in triplicate.

Supplementary Figure 3.

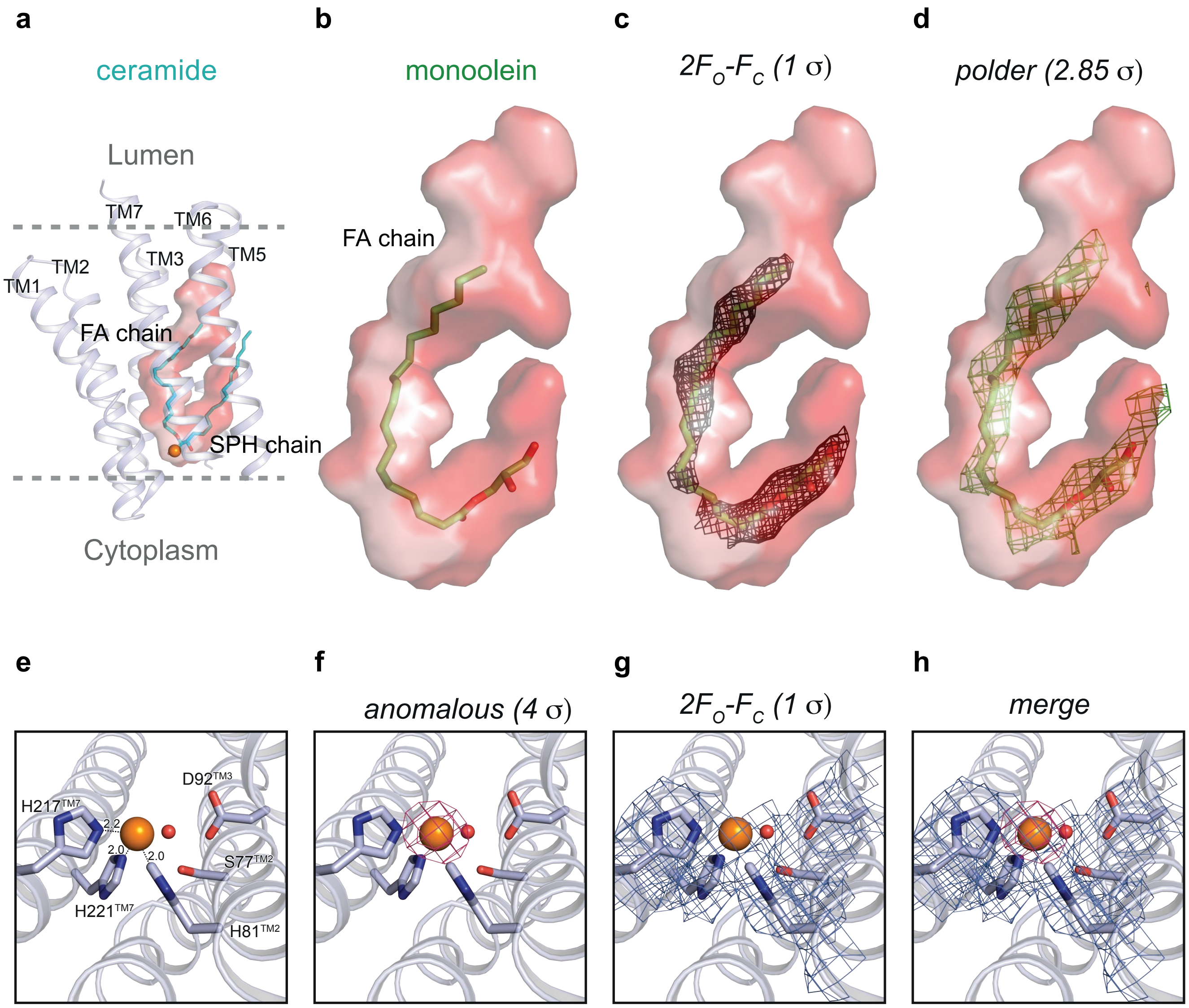

Supplementary Figure 3. Calculated electron density maps and tentatively modeled ligands.

(a) Representation of the hook-shaped cavity within the ACER3 7TM with the ceramide binding pose. **(b) (c)** Position of the modeled monoolein and representation of the calculated 2Fo-Fc map contoured at 1 σ (b) as well as the polder OMIT map (ref 21 in the main text) contoured at 2.85 σ (c). **(e)** Close-up view of the Zn^{2+} binding site in ACER3 with the anomalous difference maps of ACER3 calculated from the data set collected at Zn-edge (i.e., λ=1.281 Å) at a resolution of 3 Å and contoured at 4 σ **(f)**, the 2Fo-Fc map contoured at 1 σ **(g)** and the superposition of both maps **(h).**

Supplementary Figure 4.

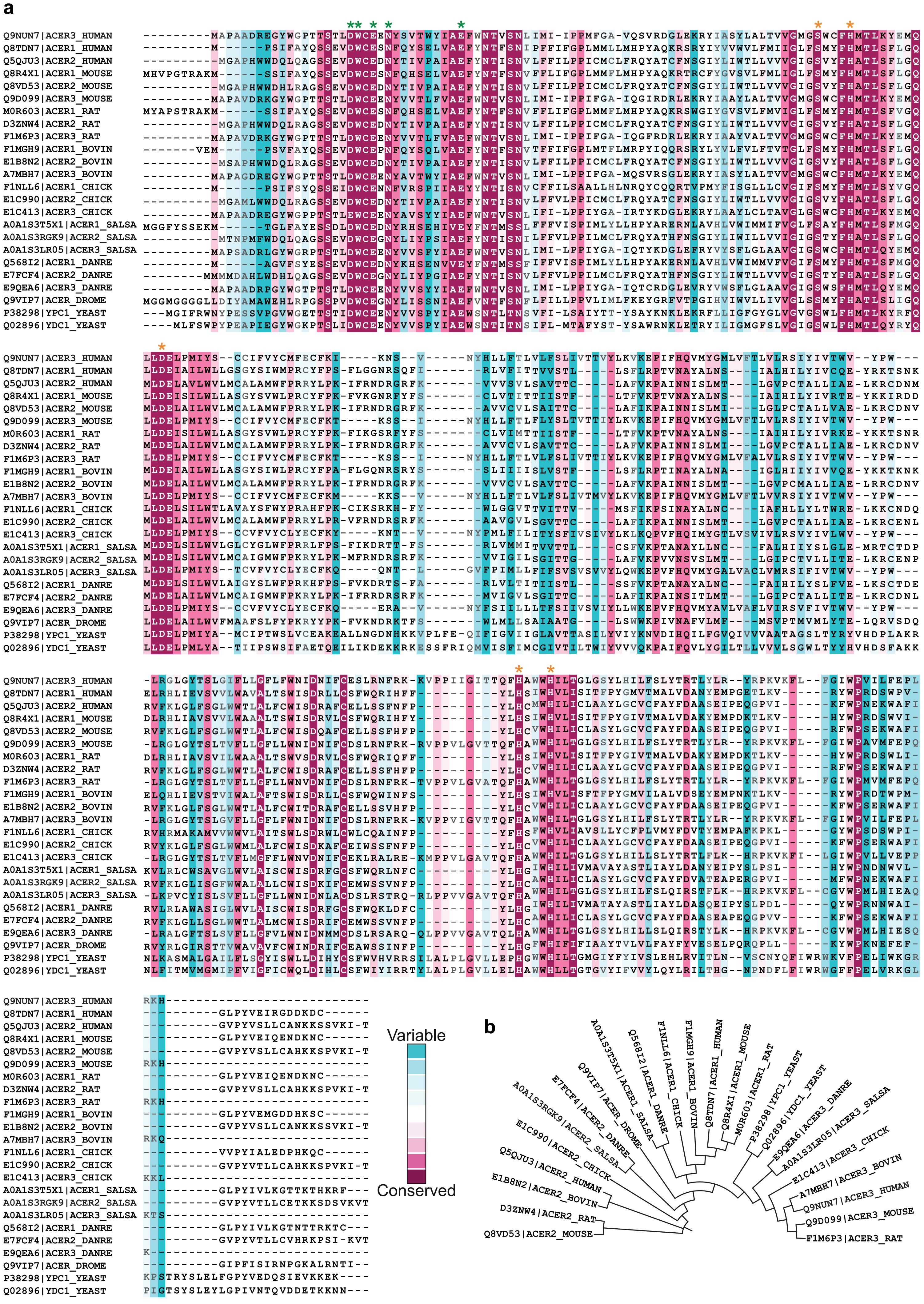

Supplementary Figure 4. ConSurf color-coded multiple sequence alignment of ACER family.

(a) Sequence alignment of alkaline ceramidases from human (Q9NUN7, Q8TDN7, Q5QJU3), mouse (Q8R4X1, Q8VD53, Q9D099), rat (M0R603, D3ZNW4, F1M6P3), bovine (F1MGH9, E1B8N2, A7MBH7), chicken (F1NNL6, E1C990, E1C413), salmon (A0A1S3T5X1, A0A1S3RGK9, A0A1S3LR05), zebrafish (Q568I2, E7FCF4, E9QEA6), fruit fly (Q9VIP7) and yeast (P38298, Q02896) created using Consurf server[1] (consurf.tau.ac.il). The color-coding bar shows coloring scheme according to degree of conservation ranging from not conserved, cyan to highly conserved, magenta. Residues coordinating Ca^{2+} (green asterisks) and Zn^{2+} (orange asterisks) ions are outstandingly conserved indicating the importance of Ca^{2+} and Zn^{2+} binding sites. **(b)** Representation of the phylogenetic tree created using Consurf server.

Supplementary Figure 5.

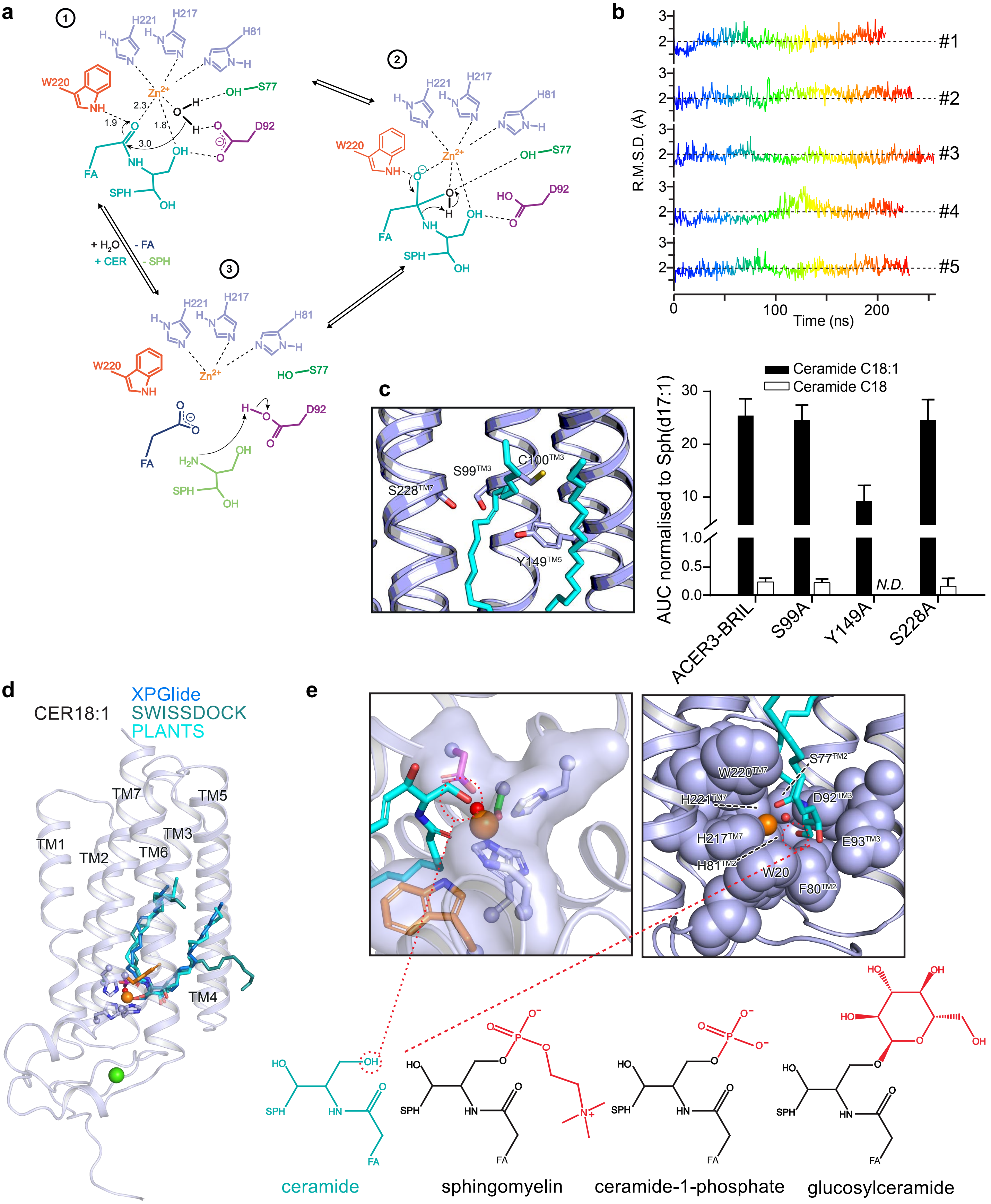

Supplementary Figure 5. Proposed general acid-base catalytic mechanism, MD simulation of C18:1 ceramide and steric hindrance for ceramide derivatives.

(a) Based on the interactions with docked ceramide C18:1 and the Zn^{2+} catalytic site architecture in the structure, we propose a general acid-base catalysis mechanism for the hydrolysis of the amide bond by ACER3. In this mechanism, the zinc ion activates a water molecule for nucleophilic attack of the amide carbon in which $D92^{TM3}$ acts as a proton acceptor/donor (1 and 3). W220 side chain polarizes the amide carbonyl and, together with the Zn^{2+}, stabilize the oxyanion formed in the tetrahedral transition state (2). **(b)** Representation of all-atom R.M.S.D. of the bound C18:1 ceramide in trajectory of five independent simulations. **(c)** Close up view of the S99, Y149, S228 domain (left panel) and enzymatic assays performed with the C18:1 (black bars) or C18 (grey bars) substrates showing the Area Under the Curve (AUC) sphingosine signal normalized to the internal standard (Sph d17:1) for ACER3-BRIL, ACER3-BRIL-S99A, ACER3-BRIL-Y149A and ACER3-BRIL-S228A mutants (right panel). The results shown are the mean ± s.d. of two independent experiments performed in pentaplicate. **(d)** Top scoring C18:1 ceramide binding poses obtained using GlideXP, the SWISSDOCK webserver (in blind docking mode) or PLANTS. **(e)** Left panel: close up view of the catalytic site with key residues shown as sticks and coloured as in (a). Right panel: W20, F80, H81 and D92 are shown as light blue spheres and are involved in the steric hindrance close to the primary alcohol of ceramide, supporting a possible mechanism for substrate selectivity.

Supplementary Figure 6.

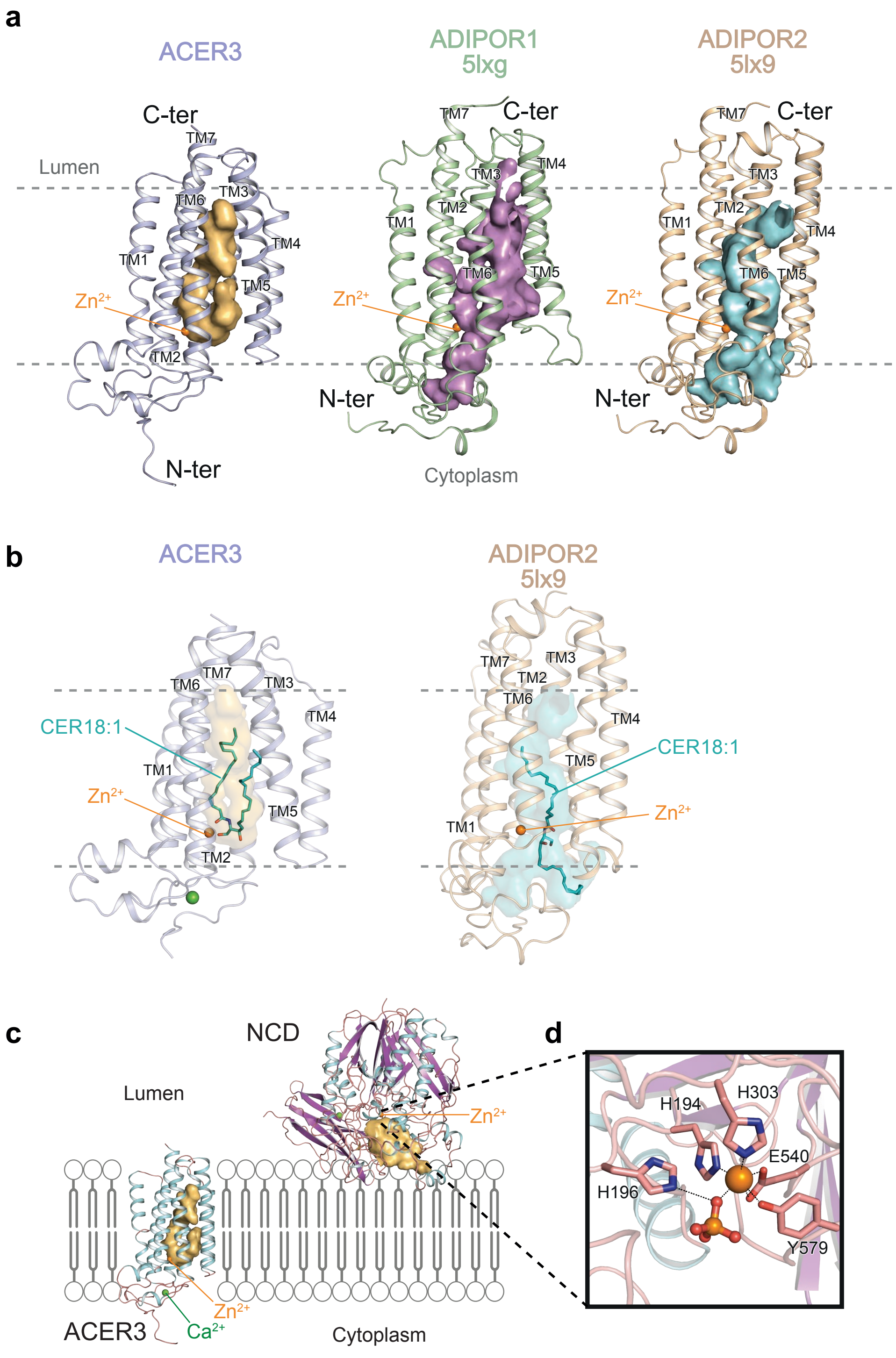

Supplementary Figure 6. Comparison of the structural features of ACER3, ADIPORs and neutral ceramidases.

(a) Structures of ACER3, ADIPOR1 and ADIPOR2 viewed from within the membrane plane highlighting the distinct architecture on the intramembrane cavities (dark yellow, blue and purple for ACER3, ADIPOR2 and ADIPOR1, respectively). **(b)** Calculated ceramide C18:1 (coloured in cyan) binding modes in ACER3 and ADIPOR2. **(c)** Comparison of the ACER3 and neutral ceramidases structures coloured according to secondary structures (α-helices in cyan and β-sheet in purple) and showing the cavity accomodating the ceramide substrate located in the lipid bilayer for ACER3 (dark yellow) in contrast to the neutral ceramidase (NCD) which contains the ceramide binding pocket outside the lipid bilayer (dark yellow). **(d)** Zoom of the Zn^{2+} catalytic site of the NCD.

Supplementary Figure 7.

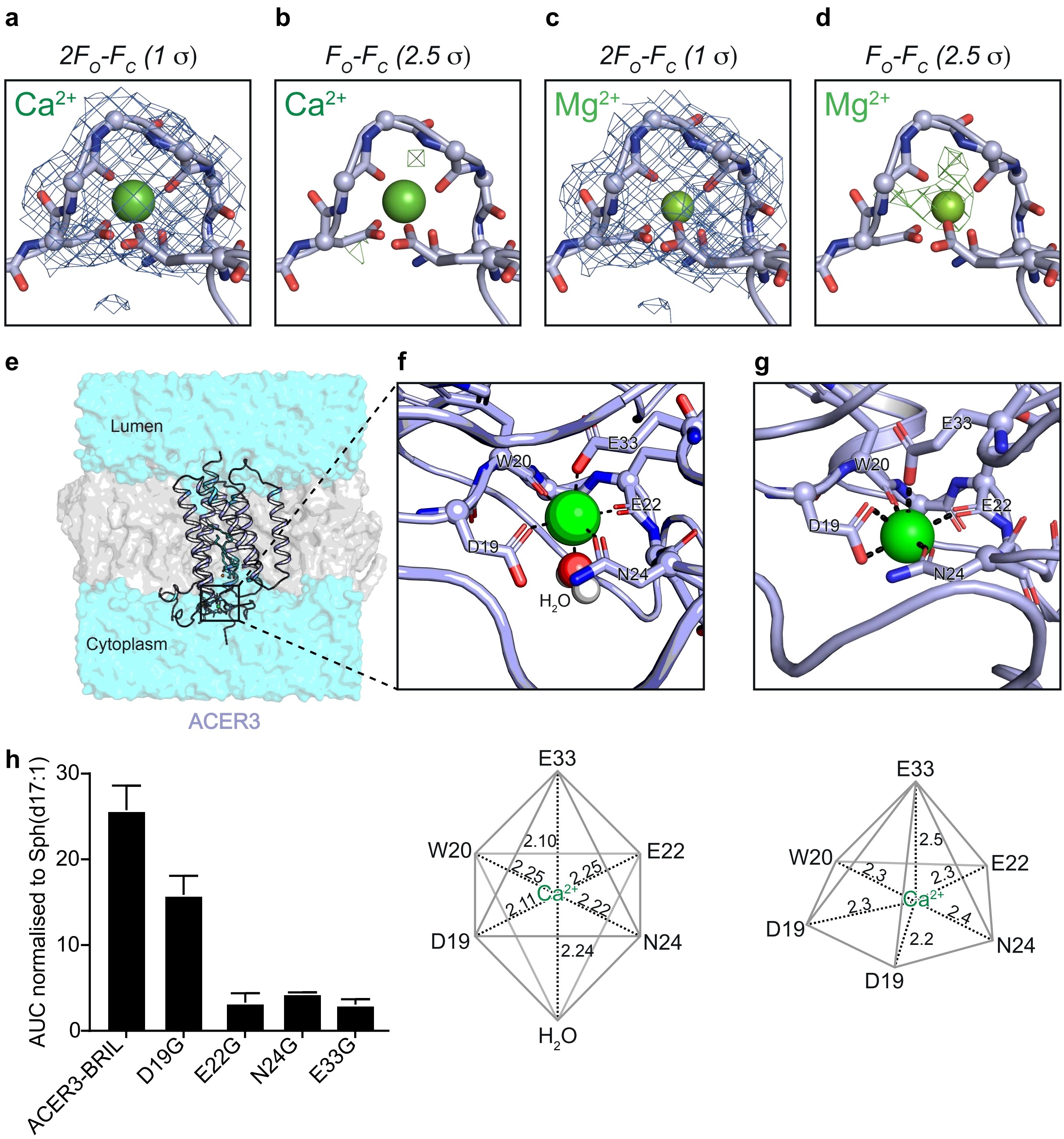

Supplementary Figure 7. Ca^{2+} coordination in the crystal structure and in MD simulations.

Comparison of 2Fo-Fc and Fo-Fc maps calculated either with Ca^{2+} **(a, b)** and Mg^{2+} **(c, d)**. The positive signal indicated as a green mesh in (d) clearly indicates that the observed electron density cannot correspond to a Mg^{2+} ion. **(e)** Overall view of ACER 3 in a lipid bilayer (grey) surrounded with water (cyan). Zoom close to the Ca^{2+} ion (green spheres) illustrating the geometry of the Ca^{2+} binding site during MD simulations (octahedral) with the water molecule shown as white and red spheres **(f)** and in the crystal structure (incomplete pentagonal bipyramid) **(g)**. **(h)** Enzymatic assays performed with the C18:1 substrate showing the Area Under the Curve (AUC) sphingosine signal normalized to the internal standard (Sph d17:1) for ACER3-BRIL, ACER3-BRIL-D19G, ACER3-BRIL-E22G, ACER3-BRIL-N24G and ACER3-BRIL-E33G mutants. The results shown are the mean ± s.d. of two independent experiments performed in pentaplicate.

Supplementary Figure 8.

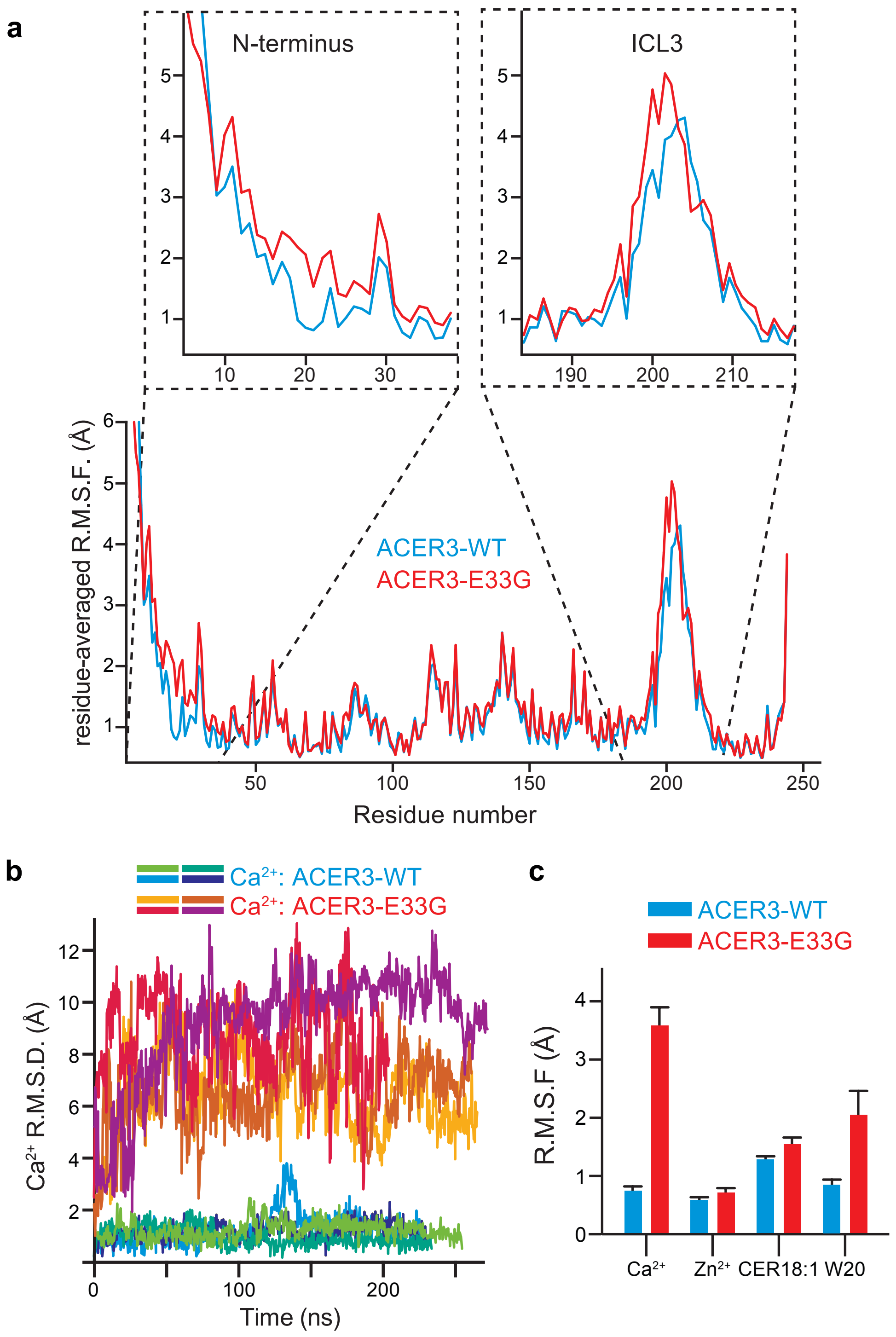

Supplementary Figure 8. MD simulations of ACER3 wild-type and E33G mutant.

(a) Residue-averaged root mean square fluctuations (R.M.S.F.) of ACER3 wild-type (blue) and E33G mutant (red), highlighting the increase in flexibility observed in the N-terminus region and ICL3. A zoom of the N-terminus and ICL3 regions are shown in inset. **(b)** Comparison of the calcium ion R.M.S.D. in five independent wild-type and mutant MD trajectories. **(c)** R.M.S.F. of selected ligands/residues in wild-type (blue) and mutant (red) MD trajectories. The error bars correspond to the standard error calculated from five independent trajectories.

Supplementary Figure 9.

a

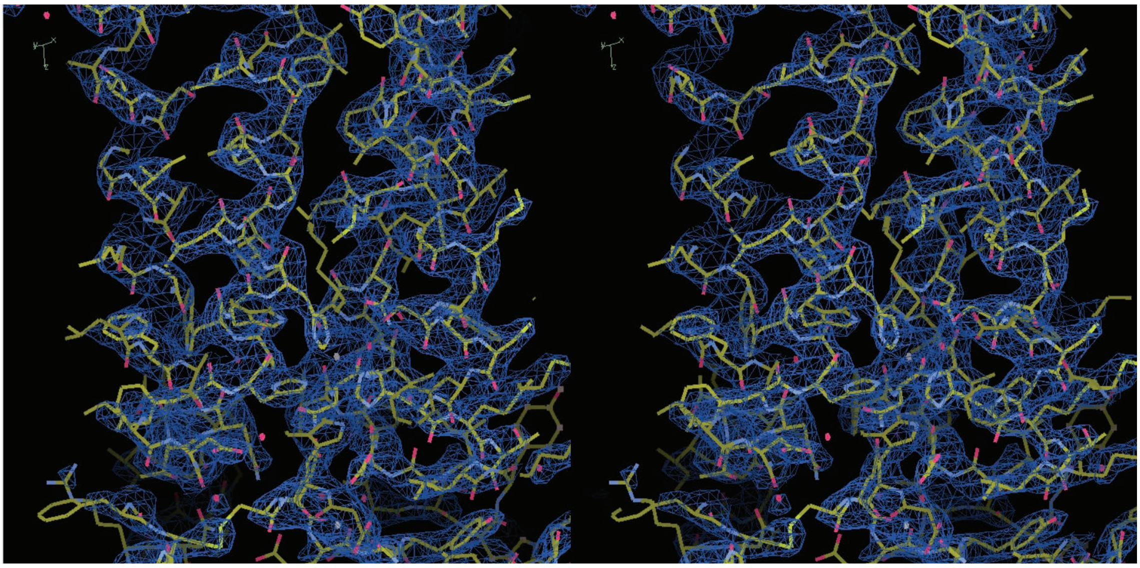

Supplementary Figure 9. Stereo images of a portion of the electron density map.

A portion of the 2Fo-Fc electron density map contoured at 1.5 σ.

Supplementary References

1 Ashkenazy, H. *et al.* ConSurf 2016: an improved methodology to estimate and visualize evolutionary conservation in macromolecules. Nucleic Acids Res **44**, W344-350, doi:10.1093/nar/gkw408 (2016).
